# Observation of Two-Step Spin Transition in Graphene Oxide-Based Hybrids with Iron(II) 4-amino-1,2,4-triazole Spin Crossover Nanoparticles

**DOI:** 10.3390/molecules28155816

**Published:** 2023-08-02

**Authors:** Nikolia Lalioti, Alexander Charitos, John Parthenios, Ondrej Malina, Michaela Polaskova, Martin Petr, Vassilis Tangoulis

**Affiliations:** 1 Laboratory of Inorganic Chemistry, Department of Chemistry, University of Patras, 26504 Patras, Greece; charitos.al97@gmail.com (A.C.); vtango@upatras.gr (V.T.); 2 Institute of Chemical Engineering Sciences (ICE-HT), Foundation for Research and Technology-Hellas (FORTH), 26504 Patras, Greece; jparthen@iceht.forth.gr; 3 Regional Centre of Advanced Technologies and Materials, Czech Advanced Technology and Research Institute (CATRIN), Palacký University Olomouc, Křížkovského 511/8, 77900 Olomouc, Czech Republic; michaela.polaskova@upol.cz (M.P.); martin.petr@upol.cz (M.P.)

**Keywords:** spin crossover, amino–triazole ligands, nanoparticles, two-step hysteresis, graphene oxide hybrids

## Abstract

A novel experimental protocol based on a reverse micellar method is presented for the synthesis of graphene oxide (GO)-based hybrids with spin crossover nanoparticles (SCO NPs) of the 1D iron(II) coordination polymer with the formula [Fe(NH_2_trz)_3_](Br_2_). By introducing different quantities of 0.5% and 1.0% of GO (according to iron(II)) into the aqueous phase, two hybrids, **NP4** and **NP5**, were synthesized, respectively. The morphological homogeneity of the NPs on the surface of the GO flakes is greatly improved in comparison to the pristine [Fe(NH_2_trz)_3_](Br_2_) NPs. From the magnetic point of view and at a low magnetic sweep rate of 1 K/min, a two-step hysteretic behavior is observed for **NP4** and **NP5**, where the onset of the low-temperature second step appeared at 40% and 30% of the HS fraction, respectively. For faster sweep rates of 5–10 K/min, the two steps from the cooling branch are progressively smeared out, and the critical temperatures observed are T_1/2_↑ = 343 K and T_1/2_↓ = 288 K, with a thermal width of 55 K for both **NP4** and **NP5**. A Raman laser power-assisted protocol was used to monitor the thermal tolerance of the hybrids, while XPS analysis revealed electronic interactions between the SCO NPs and the GO flakes.

## 1. Introduction

The study and characterization of the spin crossover (SCO) 1D coordination polymer (CP) [Fe(Htrz)(trz)_2_](BF_4_)·xH_2_O where Htrz = 1,2,4-triazole, in microscale as well as nanoscale, has been extensively documented [1,2,3,4,5,6,7,8,9,10,11,12,13,14,15,16,17,18,19,20,21,22,23]. The unique square-like hysteretic nature of the spin transition at critical temperatures well above room temperature, as well as the retaining of SCO characteristics while reducing the size of the SCO particles to values close to 10 nm, led to an extensive investigation of their candidacy as molecular switches for spintronic applications [5,6,7,8,9,10]. In the quest for technological applications, translating the center of the thermal hysteresis to temperatures close to room temperature is of utmost importance.

A promising candidate for accomplishing this goal is the understudied 1D CP [Fe(NH_2_trz)_3_](anion)_2_·xH_2_O. Prior to this study, a limited number of reports have been published concerning the nanosynthesis of the Fe/NH_2_trz system using the bromide anion, revealing that the center of the thermal hysteresis is indeed translated to lower temperatures with critical temperatures in the range 290–330 K [24,25,26,27]. In all cases, the reverse micelle method was applied, and essential changes concerning the characteristics of the thermal hysteresis of the produced NPs were revealed, depending mainly on the surfactant medium. Using the non-ionic Lauropal (playing the role of surfactant and oil medium), nanoparticles (NPs) with dimensions 30–50 nm were synthesized with a thermal hysteresis width of 2 K and critical temperatures T_1/2_↑ = 312 K and T_1/2_↓ = 310 K [24,25,26]. Recently, our group presented a reverse micellar nano-synthetic method using Triton X-100 as a surfactant, n-hexanol as a co-surfactant, and cyclohexane as the organic matrix [27]. It was found that the term ω_0_, which is the molar ratio of water to surfactant as well as the reaction time, plays an essential role in the morphology and the characteristics of the thermal hysteresis. For the first time, a two-step hysteresis is observed using a magnetic sweep rate of 1 K/min for ω_0_ = 10 and a reaction time equal to 20 h, while a drastic change to one-step hysteresis occurred by changing the reaction time to 2 days. Increasing the magnetic sweep rate to 10 K/min, a single-step hysteresis is observed with critical temperatures T_1/2_↑ = 330–335 K and T_1/2_↓ = 290–300 K.

To further investigate the SCO phenomenon of the 1D CP [Fe(NH_2_trz)_3_](Br)_2,_ we decided to monitor for the first time the role of GO as a co-reactant in the water phase and provide an efficient in situ experimental method for hybrid GO@SCO NPs. The use of graphene oxide (GO) over graphene has attracted interest in many scientific research fields due to its unique physical and chemical characteristics such as good dispersibility in water as well as other organic protic solvents, mechanical strength, and large surface area [28]. The oxidized form of graphene is graphene oxide, which contains hydrophilic functional groups on its surface, such as carboxyl, epoxy, and hydroxyl groups, resulting in its hydrophilic character. The main reason for choosing GO was its dual action potential. On the one hand, it functions as an aqueous surfactant, containing a large number of hydrophilic, such as hydroxyl and carboxyl functional groups, and hydrophobic (periodic arrangement of benzenes, sp^2^) regions. On the other hand, it could interact through hydrogen bonds between the on-surface oxygen-containing groups and the -NH_2_ side groups of the SCO NPs as well as pi–pi interactions between GO and the triazole ligands [28].

It should be mentioned that prior to this study, there are only a few publications dealing with ex situ methods of producing nanocomposites of SCO NPs with graphene or GO [29,30]. In the first case, [Fe(Htrz)(trz)_2_](BF_4_) NPs were deposited on the surface of graphene using sonication techniques. The center of the thermal hysteresis is increased to higher temperatures, revealing (a) a possible interaction of graphene with the SCO NPs, which slows down the SCO phenomenon for a percentage of SCO NPs, and (b) strengthening of interaction between SCO NPs due to aggregation phenomena [30]. In the second case, nanocomposites of [Fe(Htrz)(trz)_2_](BF_4_) NPs with GO were produced by simply mixing the components in ethanol. Apart from the GO@SCO nanocomposites, various types of rGO nanocomposites were also isolated by thermal reduction treatments of the GO@SCO nanocomposites [29].

In the present work, the GO-based hybrid SCO NPs of the 1D CP [Fe(NH_2_trz)_3_](Br)_2_ were isolated, varying the mass percentage of GO/iron(II). The morphology and dimensions of the SCO NPs were determined by TEM and PXRD measurements. The thermal hysteresis was investigated with sweep rate-dependent magnetic measurements and differential scanning calorimetry. XPS analysis was carried out to reveal possible electronic interactions between SCO NPs and GO flakes. Furthermore, a micro-Raman laser power-dependent study was carried out to monitor the SCO phenomenon and also to investigate the role of the GO substrate in the thermal tolerance of the NPs.

## 2. Results and Discussion

### 2.1. General Synthetic Aspects

We investigated for the first time the role of GO as a co-reactant in the water phase and synthesized hybrids of the type [Fe(NH_2_trz)_3_](Br)_2_·3H_2_O@GO **NP4** and **NP5**, varying the mass percentage of GO/iron(II). **NP4** and **NP5** were synthesized following the same experimental procedure described before [27], with the difference that graphene oxide (GO) was introduced into the aqueous phase containing the Fe(II) salt with a GO/Fe ratio equal to 0.5/100 for **NP4** and 1/100 for **NP5**.

**Figure 1:**
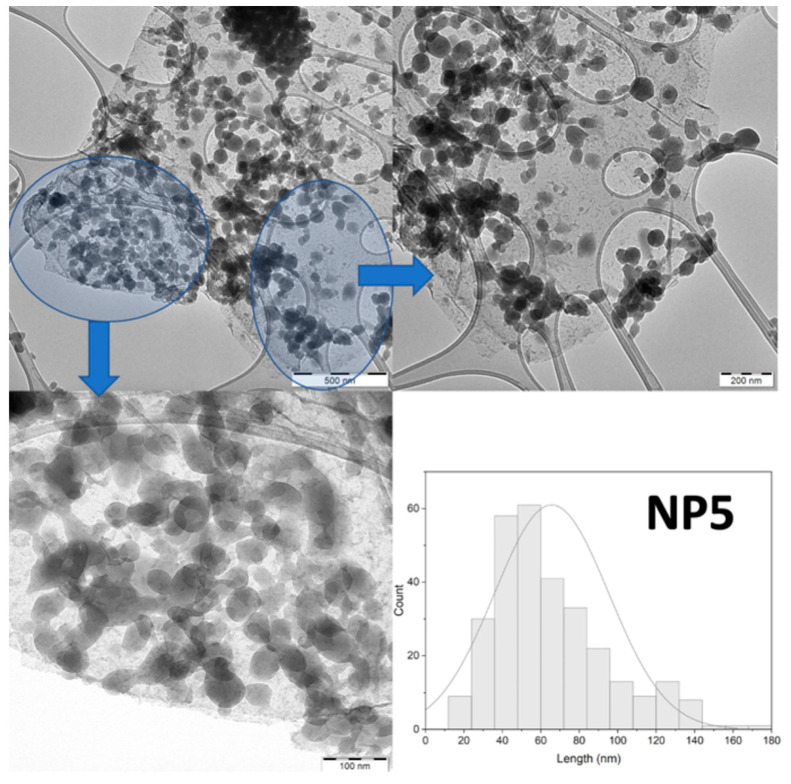
TEM images of the GO-decorated **NP5**. The highlighted regions are shown in the enlarged images guided by the arrows. The Gaussian distribution of sizes is also shown in the figure.

Transmission electron microscopy (TEM) images for **NP4** (Figure S1) reveal rectangular GO flakes uniformly covered by discoidal NPs (mean size = 80 nm), as well as scattered NPs (possibly due to the small content of GO), with heterogeneity in size and shape due to the presence of elongated plates. In the case of **NP5,** all GO layers are covered uniformly by NPs that are entirely discoidal (mean size = 70 nm), with a small number of scattered NPs (Figure 1). Saturation with the 1% GO aqueous phase is probably responsible for the smaller size and uniformity of **NP5**, which was not the case for **NP4** or the pristine [Fe(NH_2_trz)_3_](Br)_2_ NPs. Probably the combination of a sufficient amount of GO flakes and a saturated aqueous phase leads to the fact that the **NP5** particles showed the best morphological homogeneity not only in the surface of the flakes but also in the percentage of the dispersed NPs. IR spectroscopy (Figure S2, Table S1), PXRD (Figure S3), and TGA measurements (Figure S4) were carried out to characterize the final materials.

Although the crystallinity of **NP4** and **NP5** is improved considerably in comparison to the pristine [Fe(NH_2_trz)_3_](Br)_2_ NPs, it was not possible to reliably index the related diffractograms. According to our previous PXRD study, there are three 2θ regions (I, II, and III) outlining differences between the various pristine [Fe(NH_2_trz)_3_](Br)_2_ NPs (Figure S3). **NP4** and **NP5** seem to share the same characteristics as the pristine ones with synthetic parameters ω_0_ = 4 (or) 10 and a reaction time equal to 24 h [27].

The size of the crystallites was calculated using the Scherrer equation as well as the apparent size of the crystallites, $\langle L_{V}\rangle$, and the diameter of the average domain, 〈D〉, under the approximation of spherical domains. The results are shown in Table 1, along with the relevant data of the pristine [Fe(NH_2_trz)_3_](Br)_2_ NPs. It was also possible to calculate the average number of domains per particle, $\langle N_{D}\rangle$, assuming that the occupied volume of [Fe(NH_2_trz]_3_Br_2_·3H_2_O in the solid crystal is approximately 40 Å^3^ [31].

### 2.2. XPS Studies

**Figure 2:**
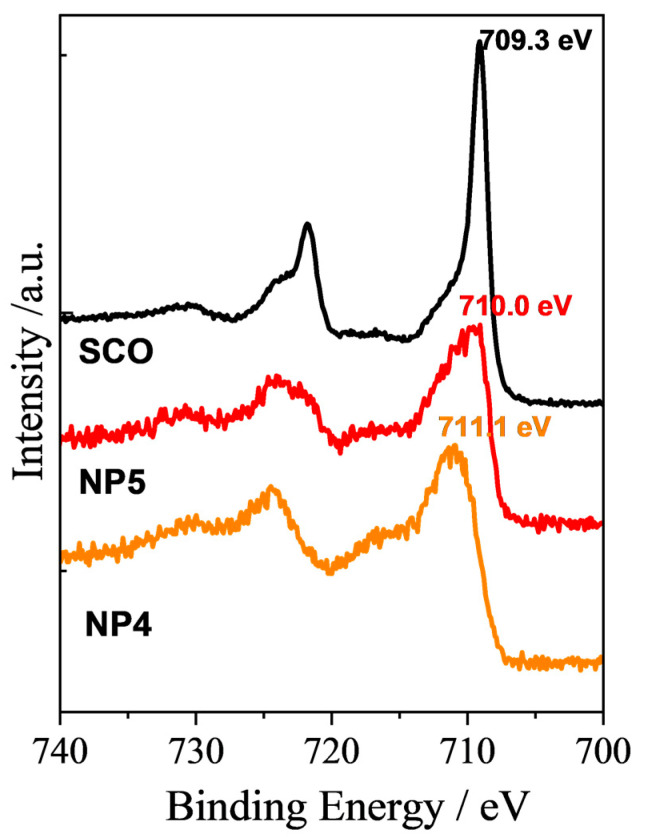
XPS spectra of Fe2p for **NP4**, **NP5,** and pristine [Fe(NH_2_trz)_3_](Br)_2_ NPs (**SCO**).

XPS analysis was employed to investigate the surface chemistry and bonding configurations of samples **NP4**, **NP5**, and pristine [Fe(NH_2_trz)_3_](Br)_2_ NPs. The Fe2p_3/2_ peak of the pristine SCO NPs is located at 709.3 eV, close to the value of 709.0 eV observed for the [Fe(Htrz)_2_(trz)](BF_4_) NPs, while a shift of 0.7 and 1.8 eV appeared for **NP4** and **NP5,** respectively (Figure 2). This shift is related to electronic interactions between the SCO NPs and the GO layer. The same behavior has been observed for the ex situ prepared nanocomposites of GO with the [Fe(Htrz)_2_(trz)](BF_4_) NPs [28].

The C*1s* peaks of **NP4** and **NP5** reveal similar peak positions and shapes, indicating comparable chemical environments and bonding configurations. At the same time, the pristine SCO NPs exhibit significant differences in the amount of C–C/C=C and C–O/C–N bonds (Figures S5 and S6). The observed peak positions and shapes of **NP4** and **NP5** are consistent with the characteristics of GO [28,32]. In contrast, the C*1s* XPS spectrum of the pristine SCO NPs exhibits a decreased intensity of C–C bonds and an increased intensity of C–O/C–N bonds, corresponding to a lower amount of oxygen and a higher amount of nitrogen (in comparison to **NP4** and **NP5**). Also, the presence of surfactant Triton X-100 should be considered [27]. XPS analysis revealed the presence of a pi–pi interaction peak in all three samples due to the sp2 carbon network and the aromatic triazole ligands.

### 2.3. Magnetic Measurements

**Figure 3:**
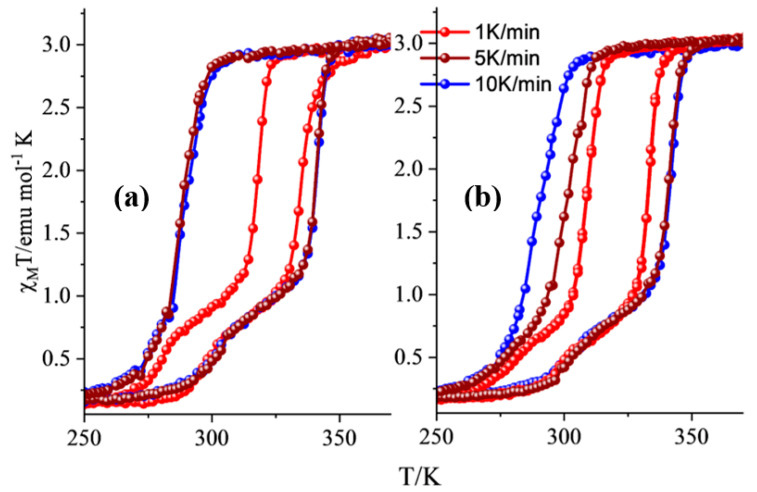
Sweep rate-dependent magnetic susceptibility measurements (third thermal cycle) carried out at 1 K/min (red solid line and spheres), 5 K/min (brown solid line and spheres), and 10 K/min (blue solid line and spheres) for (**a**) **NP4** and (**b**) **NP5** (see text for details).

A two-step experimental protocol was used for the magnetic measurements, according to which (a) a pre-heating stage took place inside the magnetometer at 400 K for 2 h to dehydrate the samples safely, and (b) three or more thermal cycles were carried out with the temperature sequence 200 K– 400 K–200 K, to ensure the stability and repeatability of the thermal hysteretic characteristics. Figure 3 shows the temperature dependence of the magnetic susceptibility (third cycle) of the dehydrated **NP4** and **NP5** at sweep rates 1 K/min, 5 K/min, and 10 K/min.

In the case of a slow magnetic sweep rate of 1 K/min, a two-step hysteresis appeared (Figure 3), according to which the high-temperature (HT) step presents critical temperatures at T_1/2_↑ = 335 K/333 K and T_1/2_↓ = 318 K/308 K, with hysteresis widths of 17 K/25 K for **NP4**/**NP5,** respectively. In comparison, the values for the second low-temperature (LT) step are T_1/2_↑=296 K/296 K and T_1/2_↓ = 283 K/281 K, with hysteresis widths of 13/15 K for **NP4/NP5**, respectively. It should be noted that similar behavior is observed for the pristine dehydrated Fe(NH_2_trz)_3_]Br_2_ NPs discussed elsewhere [27], where ω_0_ = 4 or 10 and the reaction time is equal to 20 h. A difference worth mentioning between the pristine SCO NPs and the hybrids **NP4** and **NP5** is that the onset of the second step is progressively reduced from 50% to 40% and 30% of the HS fraction, respectively. The unexpected two-step hysteretic behavior is possibly related to different polymorphs and/or the distribution of chains with different lengths [33,34]. At room temperature, the temperature-dependent susceptibility data in the form of χ_Μ_Τ is close to 3.1 emu mol^−1^ K, suggesting that the Fe(II) ions are in the HS state. In contrast, at 250 K, there is a small percentage (10%) of HS residue.

Faster magnetic sweep rates at 5 K/min and 10 K/min were also employed, and the results are shown in the same figure. The two steps are almost smeared out from the cooling branch of the hysteresis, while the second LT step in the heating branch reveals a second-order transition. More explicitly, the critical values at 10 K/min are T_1/2_↑ = 343 K and T_1/2_↓ = 288 K, with a width of 55 K for both **NP4** and **NP5,** while the second-order transition takes place in the heating mode at T = 304 K/308 K for **NP4** and **NP5**, respectively (Figure S7). The hysteresis width observed for the hybrids is the highest reported so far for the Fe/NH_2_trz system, higher than the value of 45 K presented by our group [27] for the dehydrated pristine Fe(NH_2_trz)_3_]Br_2_ NPs. Also, there is a straightforward translation of the center of the hysteresis to higher temperatures in comparison to the pristine SCO NPs, denoting the influence of the interaction of the NPs with the GO layers, which slows down the SCO phenomenon and/or increases the elastic interactions between the NPs due to possible aggregation of NPs in the GO layer. The magnetic sweep rate-dependent measurements revealed that, at high sweep rates, the heating branch is shifted to a higher critical temperature (T_1/2_↑=343 K). In contrast, the cooling component is translated to a lower value (T_1/2_↓ = 288 K), increasing the overall hysteresis width due to long metastable state lifetimes [35,36].

Differential scanning calorimetry (DSC) measurements were carried out using open crucibles to investigate the SCO phenomenon of **NP4 and NP5** in the range of 250–400 K at 10 K/min, and the results (after the 3rd thermalization cycle) are shown in Figure S3. The calculated exothermic and endothermic peaks are in close agreement with the results obtained from the magnetic measurements (Figure S8).

### 2.4. Raman Studies

**Figure 4:**
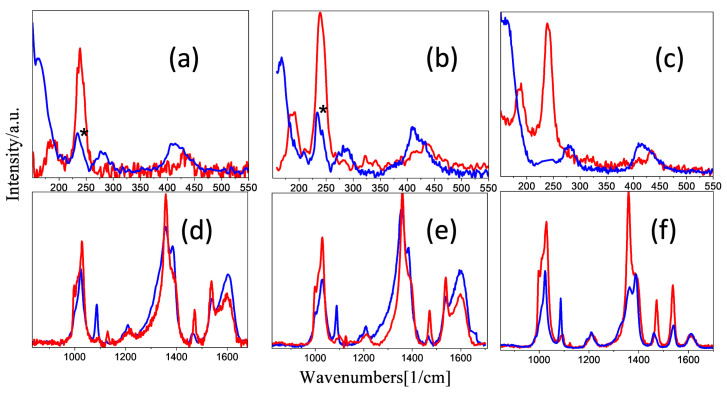
Comparison of HS Raman spectra (blue line) and LS Raman spectra (red line) in the range of 150–500 cm^−1^ and 850–1700 cm^−1^ of **NP4** (**a**,**d**), **NP5** (**b**,**e**), and pristine [Fe(NH_2_trz)_3_]Br_2_ NPs (**c**,**f**), respectively. The experimental conditions of spectra are the following: (**a**) LS spectra: laser power 84 μW, exposure time 150 s. (**b**) HS spectra: laser power 340 μW, exposure time 150 s. A percentage of the LS state is depicted in a low-intensity band at 248 cm^−1^ (presented with an asterisk), denoting the increased thermal tolerance of the hybrids.

**Figure 5:**
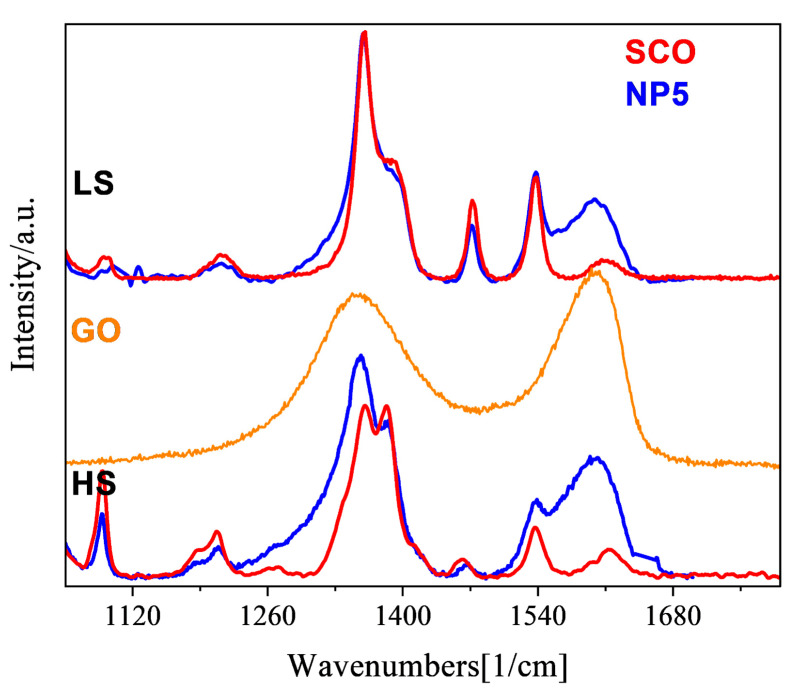
Comparison of HS/LS Raman spectra of **NP5** (blue line) and pristine [Fe(NH_2_trz)_3_]Br_2_ NPs **SCO** (red line), with GO (orange line) in the range of 1100–1700 cm^−1^.

Our group discovered that it is possible to trigger the HS and/or LS state of the [Fe(NH_2_trz)_3_]Br_2_ NPs at specific values of the laser power and exposure time [27]. More explicitly, the LS state is fully resolved using a laser power equal to 84 μW and an exposure time of 150 sec. The HS state is fully activated by increasing the laser power to 340 μW and keeping the exposure time at 150 s. The same experimental protocol was followed for **NP4** and **NP5,** and the Raman spectra in the LS/HS states are shown in Figure 4 and Figure 5, while the assignment of the peaks concerning the LS Raman spectra is defined in Table S2. The identification feature of the spin transition is represented by low-intensity bands in the range 150–300 cm^−1^, belonging to the bending and torsion vibrations of the Fe–N interaction. It was found that in the case of the Fe(NH_2_trz)_3_]Br_2_ NPs, these peaks disappear when the HS state is fully activated [27,37]. On the contrary, a percentage of the LS state remains in the HS spectra of **NP4** and **NP5,** since a low-intensity band at 248 cm^−1^ (presented with an asterisk) is resolved, denoting the increased thermal tolerance of the nanocomposites (Figure 4). Two characteristic bands of the GO product are located at 1353 cm^−1^ and 1605 cm^−1^, identified as D and G bands, respectively. The relative intensity ratio of these bands is direct evidence of the possible defects presented in the GO layer. In the Raman spectra of **NP4** and **NP5,** the D band is overlapped with two intense bands at 1368 and 1398 cm^−1^ related to the C-N stretching modes of the triazole ring, while the G band is well defined, both in the HS and LS spectra (Figure 5). For comparison reasons, the HS/LS spectra of the pristine Fe(NH_2_trz)_3_]Br_2_ NPs are also presented in the same figure.

## 3. Experimental Section

### 3.1. Materials–Instrumentation–Physical Measurements

All manipulations were performed under aerobic conditions using reagents and solvents (Alfa Aesar, Haverhill, MA, USA; Sigma Aldrich, St. Louis, MO, USA; Serva, Catoosa, OK, USA) as received. The ligand 4-amino-1,2,4-triazole (NH_2_trz) was purchased from Alfa-Aesar, the iron(II)bromide hexahydrate salt, FeBr_2_·6H_2_O, and n-hexanol from Sigma Aldrich. Triton X-100 and cyclohexane were obtained from Serva. Graphene oxide dry powder, GO, was procured from Abalonyx, Norway, product 18002: <100 mesh. The deionized water used for synthesis was deoxygenated by simultaneous sonication and argon bubbling for 1 h.

IR spectra (4000–400 cm^−1^) were recorded using a Perkin-Elmer 16PC FTIR spectrometer with samples prepared as KBr pellets.

Raman measurements were carried out using a Renishaw inVia Raman spectrometer in backscattering geometry. The beam of a solid-state 515 nm (Cobalt Fandango) laser was focused using a 50× objective lens (NA 0.75), yielding a laser spot size of about 1μm. A 2400 grooves mm^−1^ diffraction grating dispersed the Raman scattered radiation. For ease of comparison, most measurements of all samples resulted after 5 accumulations with laser powers of 85 and 340 μW, respectively, with an exposure time of the 150 s each. After acquiring the standard protocol spectra, as explained above, the thermal tolerance of each sample was examined. Increased laser power and exposure time eventually resulted in the formation of iron oxide, which was confirmed by a Raman measurement with the laser beam focused on the visibly damaged area of the sample.

The powder X-ray diffraction (PXRD) measurements were performed at room temperature on a Bruker D8 Advance diffractometer with focusing Kα1 geometry. Polycrystalline samples were loaded in 1 mm borosilicate glass capillaries while the X-ray tube operated at 45 kV and 40 mA. The incident-beam side (CuKα1 radiation, λ = 1.54056 Å) is equipped with a focusing X-ray mirror, a 0.5° fixed divergence slit, 0.5° anti-scatter slits, and 0.04 rad Soller slits, while on the diffracted-beam side, the system was configured with 0.04 rad Soller slits and a PIXcel1D detector with anti-scatter shielding. Four scans were performed in Debye-Scherrer mode, with a step size of 0.0066° on a spinning stage (~300 rpm), within a 2θ range of 4.0–90.0°. No radiation damage was observed after 5 h of measurement; therefore, all scans were merged to increase counting statistics. The broadening of the Bragg peaks, also due to instrumental reasons ($\beta_{inst}$), has been deduced from the observed integral breadth (bobs), considering the instrumental contribution is mainly of the Gaussian form, $\beta_{mat}^{2}=\beta_{obs}^{2}-\beta_{inst}^{2}$. The value of $\beta_{inst}$ was determined by using a well-characterized LaB6 powder sample in which the crystallite sizes are >1000 nm, such as the broadening of the Bragg peaks only originates from the instrument. The parameter$\beta_{inst}$ is a function of the Bragg angle, but not in the small angle region used in this study (2θ < 20ο), in which it is constant and equal to 0.1108.

TEM study was performed utilizing an FEI CM20 TEM operating at 200 kV. TEM specimens were prepared by drop-casting a 3 μL droplet of nanoparticle suspension in acetone on a carbon-coated Cu TEM grid. The size of the particles is determined by ‘‘manual counting’’ using ImageJ software, v. 1.54d (https://imagej.net, accessed on 10 June 2023).

The direct-current (DC) magnetic susceptibility measurements were measured on powder samples using a physical-properties measurement system (PPMS, Quantum Design, San Diego, CA, USA) at 200–400–200 K thermal loops with a rate of 1.0/5.0/10.0 K min^−1^ under an applied dc magnetic field of 1000 Oe. The experimental data were corrected for the diamagnetism and signal of the sample holder, and the Pascal constants were used for the diamagnetic corrections.

Differential scanning calorimetry (DSC) measurements were carried out in an N (g) atmosphere using a DSC (Q100, TA Instruments, New Castle, DE, USA) instrument. Aluminum hermetic pans encapsulated 5–7 mg of the sample. The pans were purged with nitrogen at a rate of 50 mL·min^−1^, and liquid nitrogen was used for cooling. Initially, the samples were cooled down from 253 K to 410 K at 10 K/min. Then, the samples were subjected to three successive thermal regimes of (a) heating from 253 K to 410 K at a rate of 10 K min^−1^ and (b) cooling to 253 K at the same rate. Each sample was examined with open crucibles to avoid trapping enclosed water molecules. At the beginning and at the end of each heating and cooling run, the sample was held isothermally for 5 min.

The XPS measurements were carried out with the Nexsa G2 XPS system (Thermo Fisher Scientific, Waltham, MA, USA) with a monochromatic Al-K_α_ source and photon energy of 1486.7 eV. The samples were mounted to a holder with double-sided tape (SCOTCH). All the spectra were measured in the vacuum of 1.2 × 10^−7^ Pa and at the room temperature of 20 °C. The analyzed area on each sample was the spot 400 µm in diameter. The survey spectra were measured with a pass energy of 150.00 eV and electronvolt step of 1.0 eV, while for the high-resolution spectra, a pass energy of 50.00 eV and an electronvolt step of 0.1 eV were used. Charge compensation was used for all measurements. The spectra were evaluated with the Avantage 6.5.1 (Thermo Fisher Scientific) software.

### 3.2. Preparation of Nanoparticles NP4, NP5

[Fe(NH_2_trz)_3_](Br)_2_·3H_2_O@GO, **NP5**. A saturated aqueous phase was prepared by mixing a solution of FeBr_2_·6H_2_O (216 mg, 1 mmol in 0.3 mL of deionized H_2_O) with 0.2 mL of GO suspension (2 mg, in 0.2 mL of deionized H_2_O, 30 min sonication). The aqueous phase was added to a solution containing Triton X-100 (1.8 mL), n-hexanol (1.8 mL), and cyclohexane (7.5 mL). The resulting mixture was stirred vigorously until the formation of a clear water-in-oil microemulsion. A similar procedure, but without the addition of GO, was applied to 4-amino-1,2,4-triazole (NH_2_trz) (252 mg, 3 mmol in 0.5 mL of deionized H_2_O). The ligand microemulsion was added to the GO/metal (1/100) microemulsion and the mixture was stirred for 20 h. The addition of acetone (20 mL) destabilizes the final microemulsion and promotes the precipitation of NPs. The nanoparticles were extracted by centrifugation, washed with ΕtOH (3 × 12 mL) and AcO (1 × 12 mL), and finally dried under vacuum (0.1 mbar, 24 h). Yield: 385 mg.

[Fe(NH_2_trz)_3_](Br)_2_·3H_2_O@GO, **NP4**. An aqueous phase was prepared by mixing a solution of FeBr_2_·6H_2_O (216 mg, 1 mmol in 0.3 mL of deionized H_2_O) with 0.1 mL of GO suspension (1 mg, in 0.2 mL of deionized H_2_O, 30 min sonication). The aqueous phase was added to a solution containing Triton X-100 (1.8 mL), n-hexanol (1.8 mL), and cyclohexane (7.5 mL). The resulting mixture was stirred vigorously until the formation of a clear water-in-oil microemulsion. A similar procedure, but without the addition of GO, was applied to 4-amino-1,2,4-triazole (NH_2_trz) (252 mg, 3 mmol in 0.5 mL of deionized H_2_O). The ligand microemulsion was added to the GO/metal (0.5/100) microemulsion, and the mixture was stirred for 20 h. The addition of acetone (20 mL) destabilizes the final microemulsion and promotes the precipitation of NPs. The nanoparticles were extracted by centrifugation, washed with ΕtOH (3 × 12 mL) and AcO (1 × 12 mL), and finally dried under vacuum (0.1 mbar, 24 h). Yield: 362 mg

## 4. Conclusions

A new synthetic approach is presented for the synthesis of GO-based hybrids of spin crossover nanoparticles (SCO NPs) of the 1D iron(II) coordination polymer with the formula [Fe(NH_2_trz)_3_](Br_2_). The GO flakes are in situ introduced in the aqueous phase containing the Fe(II) ions, and two products were isolated with a GO/Fe ratio equal to 0.5/100 for **NP4** and 1/100 for **NP5**. The magnetic sweep rate dependence of the susceptibility data revealed that at the slow rate of 1 K/min, two-step hysteretic phenomena appeared where the onset of the LT second step is observed at 40% and 30% of the HS fraction for **NP4** and **NP5**, respectively. By increasing the sweep rate to values 5–10 K/min, the two steps are progressively smeared out. While the abrupt cooling branch is shifted to a lower critical temperature T_1/2_↑ = 288 K, the heating branch is translated to a higher critical temperature T_1/2_↑ = 343 K, giving rise to a thermal width of ΔΤ = 55 K. The hysteresis width observed for the hybrids is the highest reported so far for the Fe/NH_2_trz system with the cooling branch located in ambient temperatures.

The influence of the GO in the hybrids is manifested from (a) the morphological homogeneity of the NPs in the surface of the GO flakes, (b) the increase of the thermal width of the hysteresis by 10 K, compared to the pristine [Fe(NH_2_trz)_3_](Br)_2_ NPs revealing long metastable state lifetimes, (c) their thermal tolerance according to the Raman studies, (d) the shift of the Fe2p_3/2_ peak to higher energy values in comparison to the pristine NPs denoting the electronic interaction of the SCO NPs with the GO layers. Further investigation is underway on the role of GO and GO derivatives as co-reactants in reverse micellar methods for synthesizing SCO@GO materials.

## Figures and Tables

**Table 1:** Average distribution sizes of the studied SCO NPs derived from TEM measurements, the calculated average apparent domain size, $\langle L_{V}\rangle$, and the diameter of the coherent domains, 〈D〉, from PXRD measurements and the average number of domains per nanoparticle, $\langle N_{D}\rangle$.

NPs	Distribution Size [nm]	$\langle L_{V}\rangle \;[\mathrm{nm}]$	〈D〉	$\langle N_{D}\rangle$
NP1 [27]	70	19	13	160
NP2 [27]	70	8	10	350
NP3 [27]	60	18	24	16
NP4	80	10	13	240
NP5	70	20	26	20

**NP4** follows the general trend described by our group and others, according to which its size is inversely proportional to the diameter of the coherent domain [25,27]. An exception exists in the case of **NP5**, denoting the influence of the GO layer.

## Data Availability

Not applicable.

## Supplementary Materials

The following supporting information can be downloaded at https://www.mdpi.com/article/10.3390/molecules28155816/s1, Figure S1: TEM images of **NP4** with Gaussian distributions of sizes; Figure S2: IR spectra of **NP3–4** and pristine [Fe(NH_2_trz)_3_](Br)_2_ NPs (**SCO**); Table S1: Assignment of the observed bands in the IR spectra; Figure S3: PXRD spectra of **NP4** and **NP5**. For comparison reasons the pXRD of the pristine [Fe(NH_2_trz)_3_](Br)_2_ NPs discussed in Ref. [27] are also presented. The three distinctive areas are outlined in blue color and named I, II, III. The crystallinity of the products **NP4–5** is improved in comparison to the **NP1–3**. pXRD spectra of the NP4 and NP5 present similar characteristics with the pristine ones with synthetic parameters ω_0_ = 4 (or) 10 and a reaction time equal to 24 h (**NP1–2**) while clear differences appear in comparison to the pristine ones with synthetic parameters ω_0_ = 10 and a reaction time equal to 2 days (**NP3**); Figure S4: Thermogravimetric curves of**NP4**, **NP5,** and pristine [Fe(NH_2_trz)_3_](Br)_2_ NPs (**SCO**); Figure S5: (a) C 1s bonds quantification (b) C 1s spectrum of **NP4** (c) C 1s spectrum of **NP5** (d) C 1s spectrum of pristine [Fe(NH_2_trz)_3_](Br)_2_ NPs (**SCO**); Figure S6: Elemental quantification according to the XPS analysis of **NP4**, **NP5,** and pristine [Fe(NH_2_trz)_3_](Br)_2_ NPs (**SCO**); Figure S7: The first derivatives of the thermal hysteresis of **NP4** and **NP5** for magnetic sweep rate of 10 K/min. In the case of **NP5**, the red line is associated with the heating mode and the black line with the cooling mode. In the case of **NP4**, the red line is associated with the cooling mode and the black line with the heating mode; Figure S8: DSC curves of **NP4–5** presenting the 3rd heating–cooling cycle at 10 K/min; Table S2: Peak assignments of [Fe(NH_2_trz)_3_]Br_2_ nanoparticles in the diamagnetic low spin state(s = 0). Meaning of symbols: L, librations; T, translations; ν: stretching; δ: deformation or in-plane bending; ω, wagging; τ: torsion; rg: ring).

## Sample Availability

Samples of the compounds are available from the authors.
