# Supplementary material for: Observation of Two-Step Spin Transition in Graphene Oxide-Based Hybrids with Iron(II) 4-amino-1,2,4-triazole Spin Crossover Nanoparticles
Source: Molecules. 2023 Aug 2;28(15):5816. doi: 10.3390/molecules28155816 (PMC10421334; doi:10.3390/molecules28155816)
Supplement: Supplementary file 1 [file molecules-28-05816-s001.zip › molecules-2517426-supplementary.pdf]

Supporting Information

Observation of two-step spin transition in Graphene Oxide based hybrids with iron(II) 4-amino-1,2,4-triazole spin crossover nanoparticles

Nikolia Lalioti[1*], Alexander Charitos[1], John Parthenios[2], Ondrej Malina[3*], Michaela Polaskova[3], Martin Petr[3], Vassilis Tangoulis[1]

[1] Department of Chemistry, Laboratory of Inorganic Chemistry, University of Patras, 26504 Patras, Greece; (NL) lali@upatras.gr; (VT) vtango@upatras.gr
[2] Institute of Chemical Engineering Sciences (ICE-HT), Foundation for Research and Technology-Hellas (FORTH), 26504 Patras, Greece;
[3] Regional Centre of Advanced Technologies and Materials, Czech Advanced Technology and Research Institute (CATRIN), Palacký University Olomouc, Křížkovského 511/8, 77900, Czech Republic; (O.M.) ondrej.malina@upol.cz ; (M.P.) michaela.polaskova@upol.cz ; (M.P.) martin.petr@upol.cz;

*Correspondence: (N.L.) lali@upatras.gr; (O.M.) ondrej.malina@upol.cz ;

Table of Contents

TEM Study 2
IR Spectra 3
p-XRD measurements 4
TGA Studies 5
XPS Studies 6
Magnetic Susceptibility data at sweep rate 10 K/min 7
DSC studies 9
Raman spectroscopy 9

TEM Study

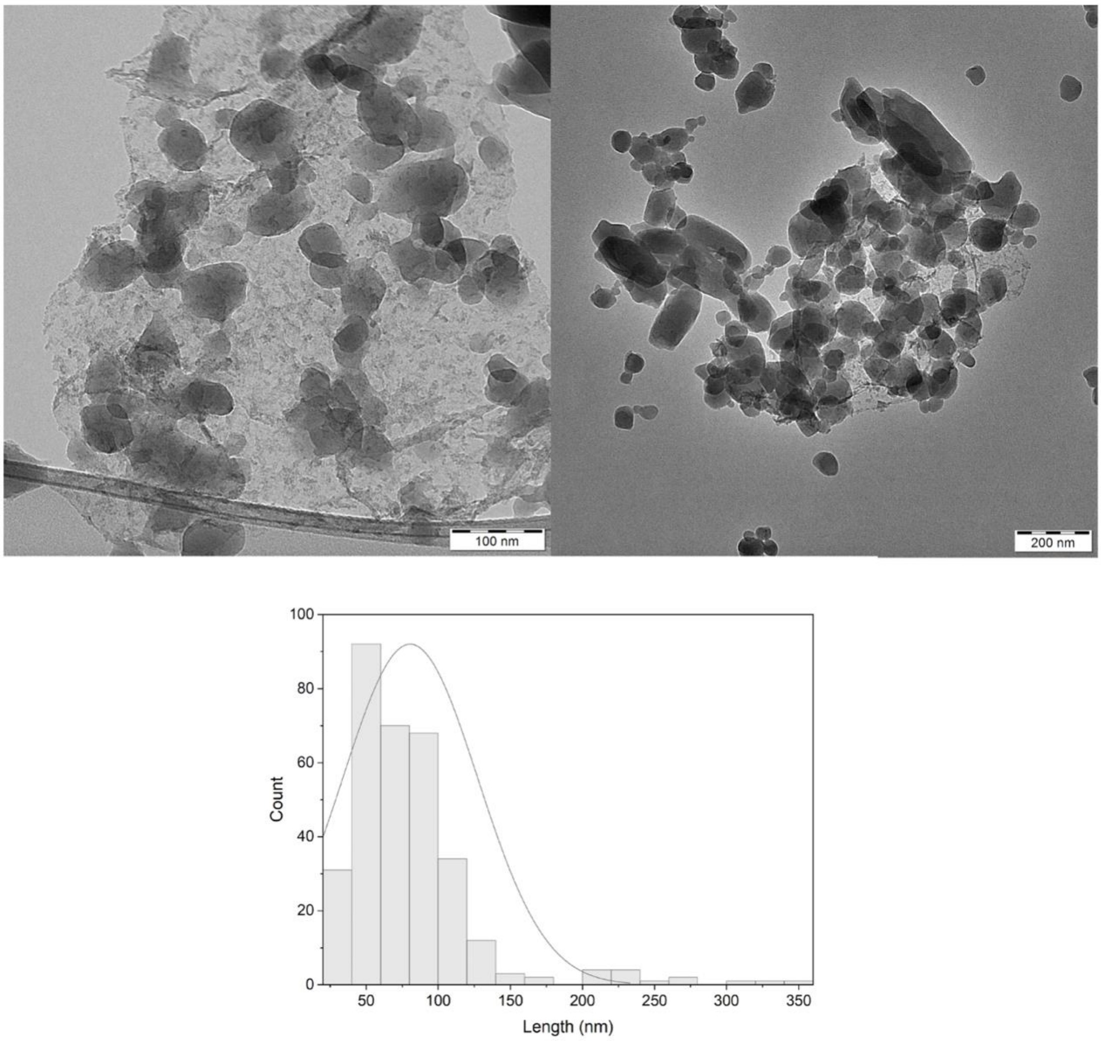

Figure S1. TEM images of **NP4** with Gaussian distributions of sizes.

IR Spectra

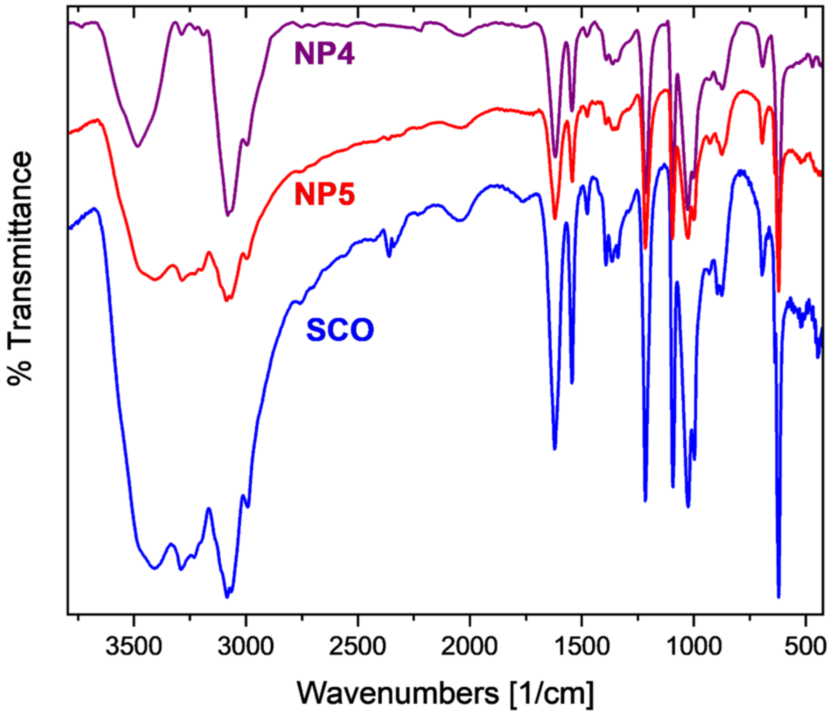

Figure S2. IR spectra of the **NP4-5** and pristine $[Fe(NH_2trz)_3](Br)_2$ NPs (**SCO).** Due to the low amount of GO in the hybrids it was not possible to identify characteristic band of the GO.

Table S1. Assignment of the observed bands in the IR spectra.

$[Fe(atrz)_3](Br)_2$	description,	$Fe(atrz)_3](Br)_2$	description,
3410s-3285 s, br	Vas(NH_2) Vs(NH_2) v(OH)water	1093s	δ(CH)
3085s	V(CH) stretch	1026s	R_6 ring stretch
1620s	δ(NH_2)	1000m	R_7 ring breathing
1545s	R_1 ring stretch	891m, br	γ(CH) bend
1475m	R_2 ring stretch	876m	β(NH_2)
1390m, br	R_3 ring stretch	693m	R_8 ring torsion
1215s	V(N-NH_2)	621s	R_9 ring torsion

m = medium, w = weak, br = broad, and s = strong

p-XRD measurements

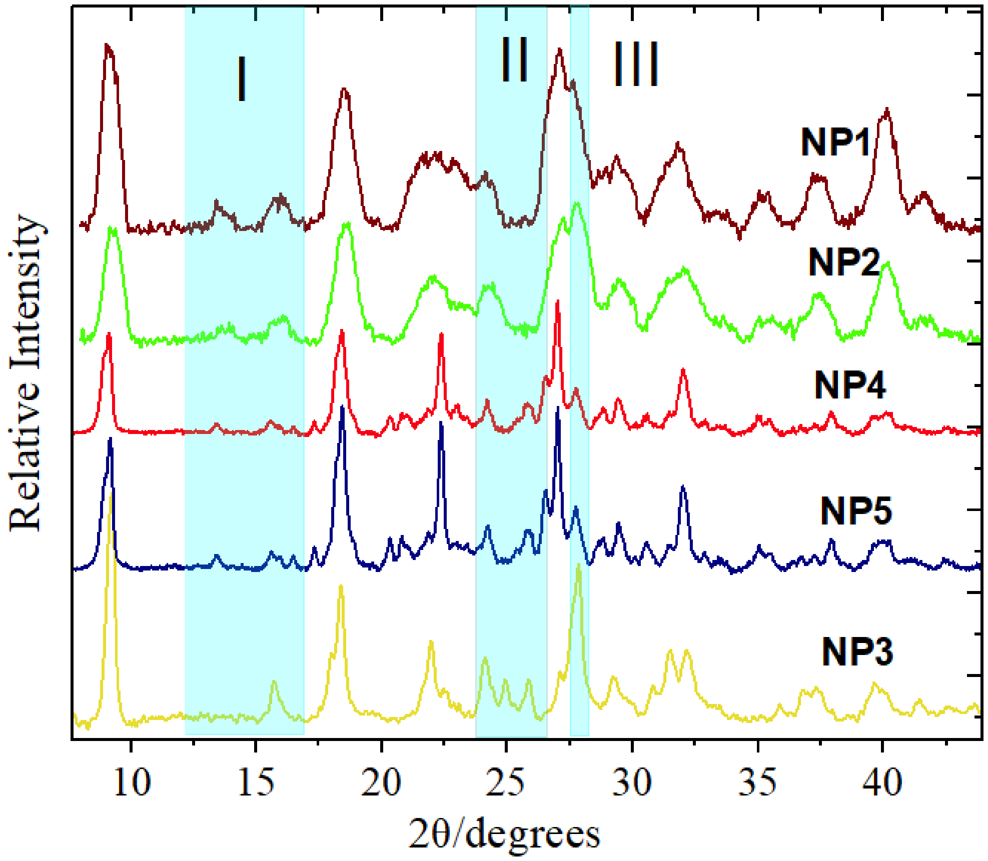

Figure S3. pXRD spectra of the **NP4** and **NP5.** For comparison reasons the pXRD of the pristine $[Fe(NH_2trz)_3](Br)_2$ NPs discussed in ref [27] are also presented. The three distinctive areas are outlined in blue color and named I, II, III. The crystallinity of the products **NP4-5** is improved in comparison to the **NP1-3.** pXRD spectra of the **NP4** and **NP5** present similar characteristics with the pristine ones with synthetic parameters ω_0= 4 (or) 10 and a reaction time equal to 24 hours (**NP1-2**) while clear differences appear in comparison to the the pristine ones with synthetic parameters ω_0= 10 and a reaction time equal to 2 days (**NP3**)

TGA Studies

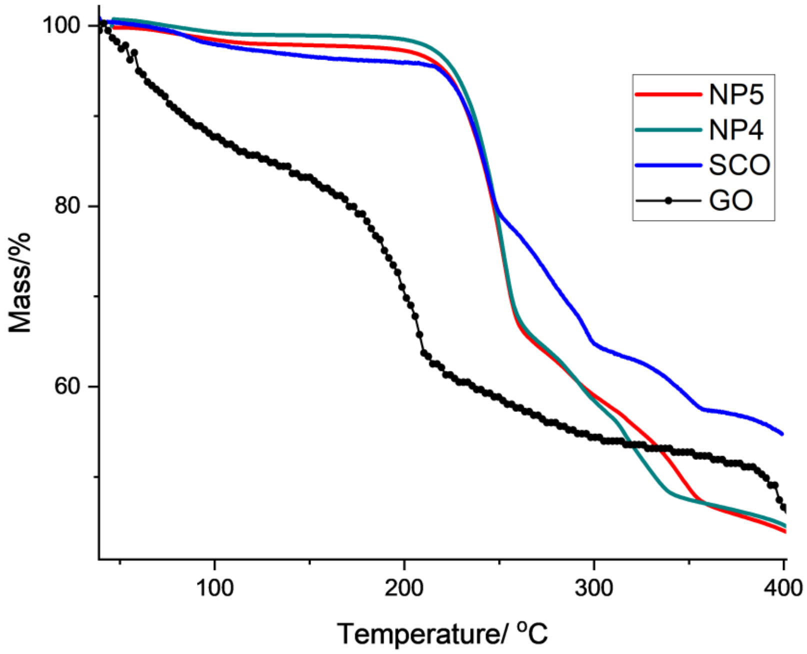

Figure S4. Thermogravimetric curves of the **NP4, NP5,** pristine [Fe(NH_2trz)$_3$](Br)$_2$ NPs (**SCO)** and **GO**. Both the hybrids and the pristine NPs are stable up to 220° C and a more abrupt decrease is monitored for the case of **NP4**, **NP5** due to their hybrid nature. Analogous behavior has been observed in the nanocomposites of [Fe(Htrz)(trz)$_2$](BF_4) NPs with GO in ref [28]. According to the thermogravimetric analysis, the initial degradation of GO until 200 ° C may be ascribed to the evaporation of CO, CO_2 and water moisture from the sheets. A slight change in the thermal decomposition in GO hybrid samples and the pristine SCO is observed, indicating that the amount of GO flakes is small to abrupt change the decomposition behaviour of the hybrids.

XPS Studies

(**a**) (**b**)

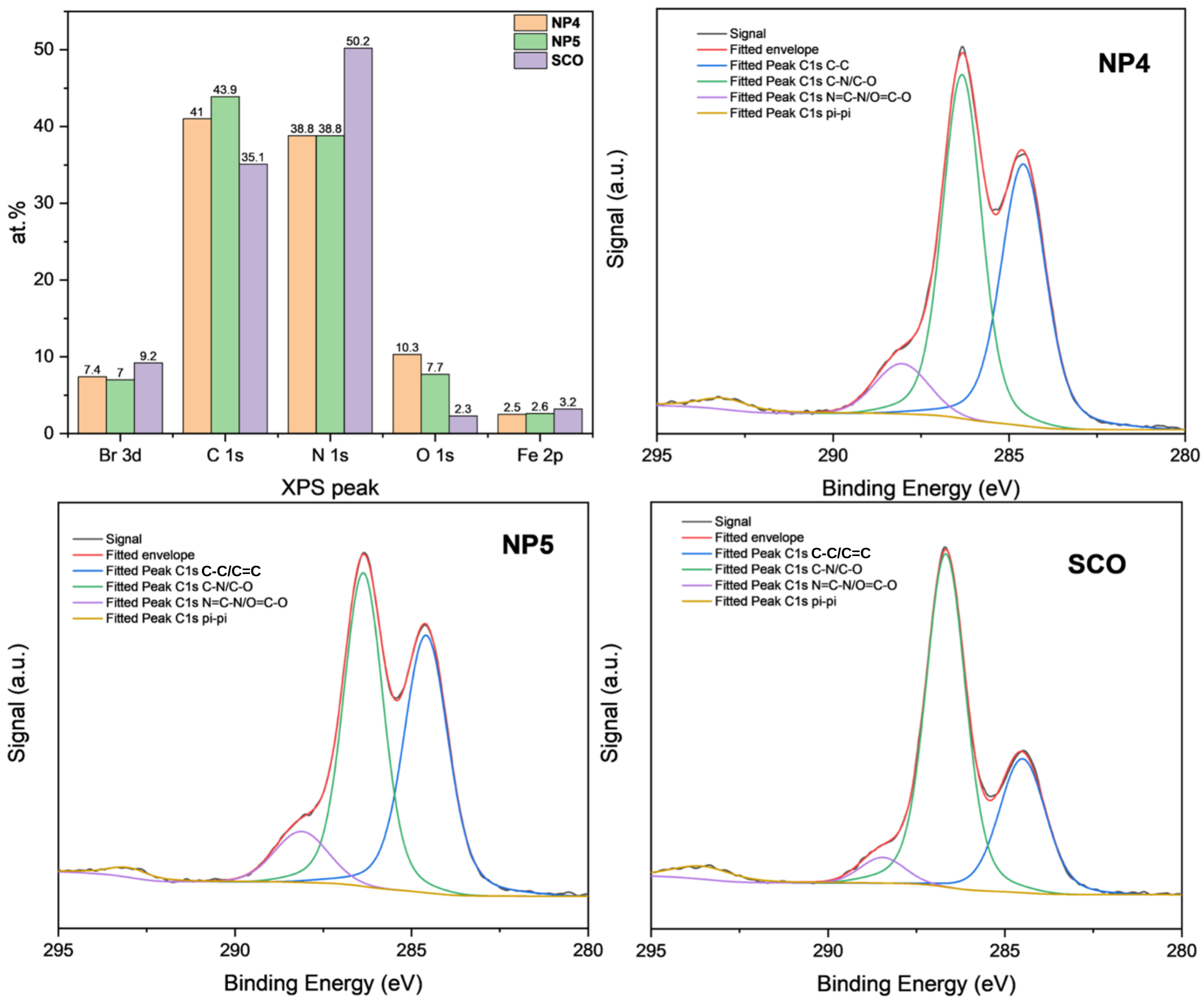

(c) (d)

Figure S5. a) C 1s bonds quantification b) C 1s spectrum of **NP4** c) C 1s spectrum of **NP5** d) C 1s spectrum of pristine [Fe(NH_2trz)$_3$](Br)$_2$ NPs (**SCO).**

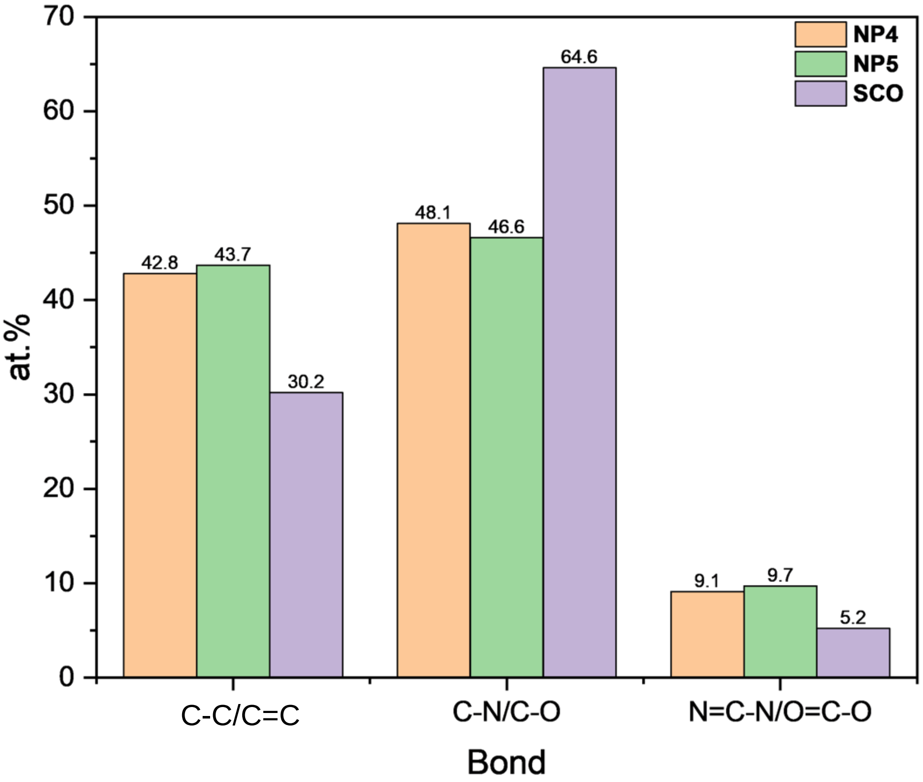

Figure S6. Elemental quantification according to the XPS analysis of the **NP4**, **NP5** and pristine $[Fe(NH_2trz)_3](Br)_2$ NPs (**SCO)**.

Magnetic Susceptibility data at sweep rate 10 K/min

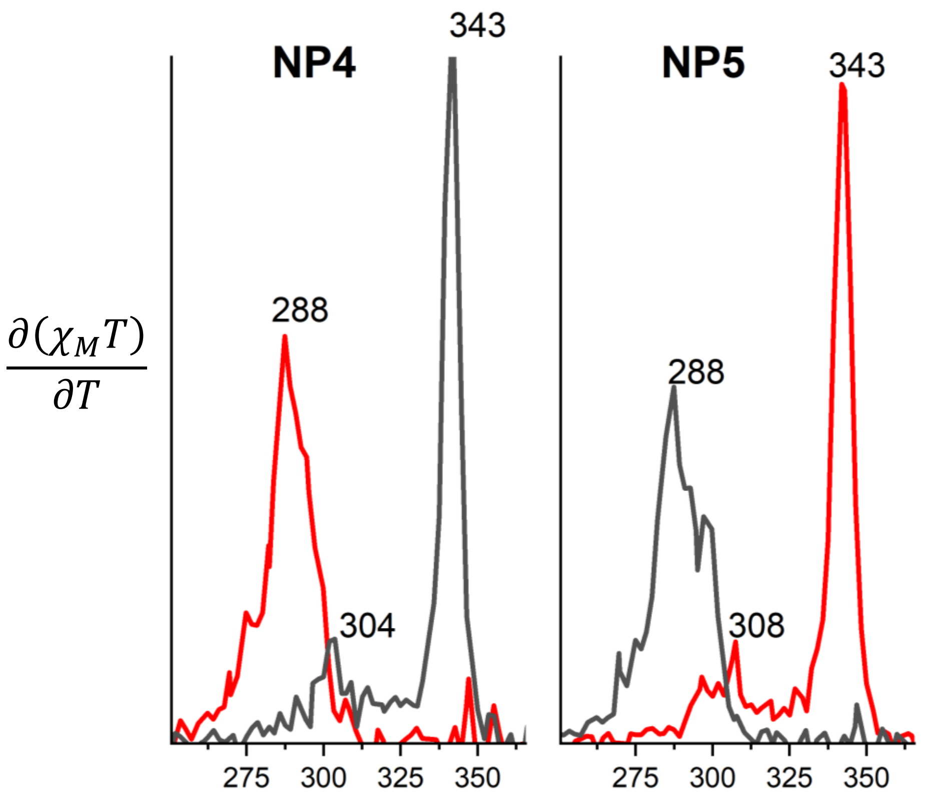

Figure S7. The first derivatives of the thermal hysteresis of **NP4** and **NP5** for magnetic sweep rate of 10 K/min. For the case of **NP5** the red line is associated with the heating mode and the black line with the cooling mode. For the case of **NP4 the** red line is associated with the cooling mode while the black line with the heating mode.

DSC studies

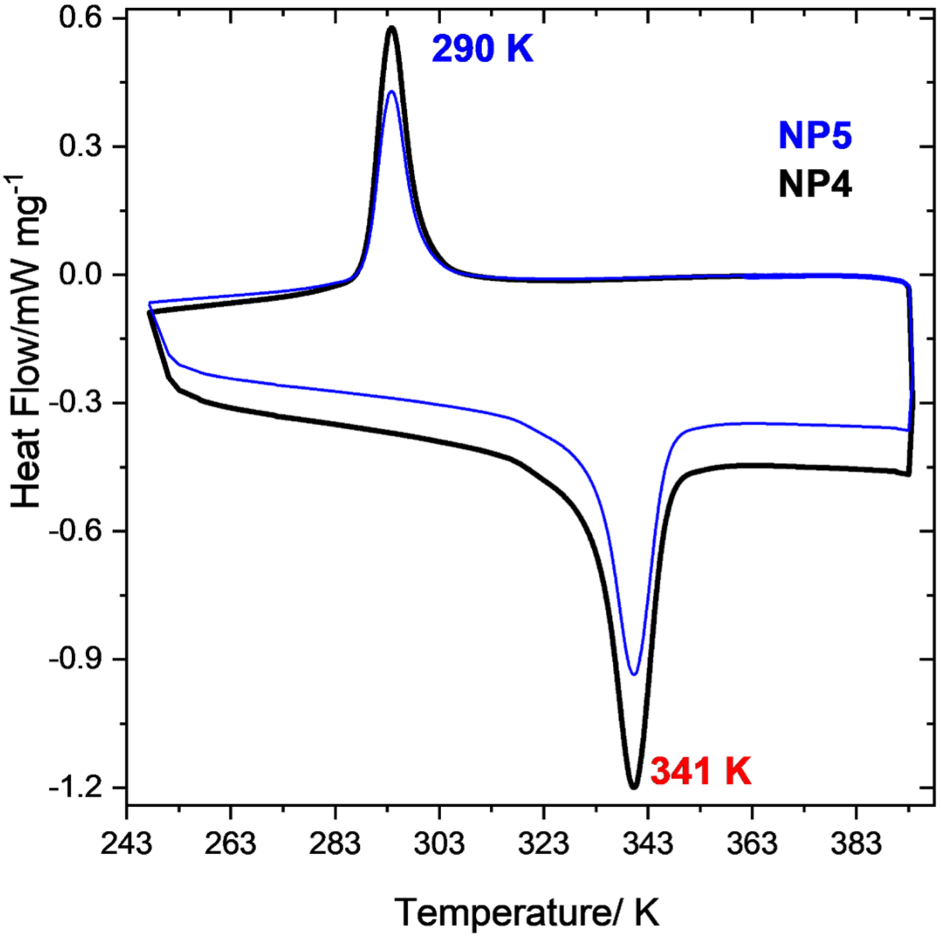

Figure S8. DSC curves of **NP4-5** presenting the 3rd heating-cooling cycle at 10 K/min.

Raman spectroscopy

Table S2. Peak assignments of $[Fe(NH_2trz)_3]Br_2$ nanoparticles (NPs **1-5**) in the diamagnetic low spin state(s=0). Meaning of symbols: L, librations; T, translations; ν: stretching; δ: deformation or in-plane bending; ω, wagging; τ: torsion; *rg*: ring)

Appx. wavenumber region (cm^{-1})	Peak assignment	Vibrational mode
195	NH_2-trz	L
248	NH_2-tz	T
445	C-N-NH_2	δ
635	NH_2	ω

702	$N-NH_2$	ν, τrg
1006	N-N	ν
1037	N-N	δrg
1104	$C-NNH_2$, H-C-N	δrg, ν, δ
1218	H-C-N	δ
1368	$C-NNH_2$	ν
1398	C-N, $N-NH_2$	ν, ν
1478	C-N	ν
1545	C-N, $C-NNH_2$, H-C-N	ν, ν, δ
1616	NH_2	δ
